# 1D/2D van der Waals Heterojunctions Composed of Carbon Nanotubes and a GeSe Monolayer

**DOI:** 10.3390/nano11061565

**Published:** 2021-06-14

**Authors:** Yuliang Mao, Zheng Guo, Jianmei Yuan, Tao Sun

**Affiliations:** 1 Hunan Key Laboratory for Micro–Nano Energy Materials and Devices, School of Physics and Optoelectronic, Xiangtan University, Xiangtan 411105, China; 201821521377@smail.xtu.edu.cn; 2 Hunan Key Laboratory for Computation and Simulation in Science and Engineering, School of Mathematics and Computational Science, Xiangtan University, Xiangtan 411105, China; 202021001381@smail.xtu.edu.cn

**Keywords:** germanium selenide, carbon nanotubes, heterojunction, photoelectric conversion efficiency

## Abstract

Based on first-principles calculations, we propose van der Waals (vdW) heterojunctions composed of one-dimensional carbon nanotubes (CNTs) and two-dimensional GeSe. Our calculations show that (*n*,0)CNT/GeSe (*n* = 5–11) heterojunctions are stable through weak vdW interactions. Among these heterojunctions, (*n*,0)CNT/GeSe (*n* = 5–7) exhibit metallic properties, while (*n*,0)CNT/GeSe (*n* = 8–11) have a small bandgap, lower than 0.8 eV. The absorption coefficient of (*n*,0)CNT/GeSe (*n* = 8–11) in the ultraviolet and infrared regions is around 10^5^ cm^−1^. Specifically, we found that (11,0)CNT/GeSe exhibits type-II band alignment and has a high photoelectric conversion efficiency of 17.29%, which suggests prospective applications in photoelectronics.

## 1. Introduction

As a fascinating carbon material, single-wall carbon nanotubes (SWCNTs) [1] have attracted widespread attention due to their unique properties [2,3,4]. The outstanding physical properties of CNTs make them a good candidate basic material for next-generation electronic devices [3,4]. The accurate prediction of CNTs’ electronic properties is very important for their possible applications [5]. For large-diameter CNTs, the CNTs are metals or semiconductors depending on their chiral indices (*n*,*m*) [6]. When (*n* − *m*) is equal to 3*p*, where *p* is an integer, the CNT is a metal. Otherwise, the CNT is a semiconductor [6]. The above criterion is not applicable to CNTs with small diameters due to curvature effects or s-p rehybridization [7,8,9,10,11,12,13,14,15].

Monolayer germanium selenide (GeSe) is a semiconductor that has a direct bandgap [16]. Few-layer GeSe, including monolayer GeSe, is non-toxic and can exist stably at room temperature [17,18]. Our group successfully prepared a single layer of GeSe using mechanical stripping and laser-thinning technology [19]. First-principles studies, combined with photoluminescence spectra, proved that a direct bandgap exists for less than three layers in a few-layer GeSe [20]. Under conditions of high temperature and high pressure, the GeSe conductivity is higher than that of black phosphorus and graphene [21]. Our previous study showed that monolayer GeSe, with point defect engineering, has a good adsorption effect on toxic gases [22]. Moreover, we found that the bandgap can be tuned by stacking order and external strain in bilayer GeSe [23]. Our designed GeSe/SnSe heterojunction, based on first principles, exhibited a superior photoelectric conversion efficiency (PCE) of 21.47% [24].

Duan et al. [25] recently proposed a state-of-the-art material design called van der Waals (vdW) integration [25]. They suggest combining a two-dimensional (2D) material with materials with other dimensions. For example, 2D and one-dimensional (1D) materials could be combined by the vdW interaction. The literature reported that a 2D/2D GeSe/SnS heterojunction has stronger optical absorption than GeSe or SnS [24]. A 0D/2D photodiode was proposed by integrating quantum dots or plasma nanoparticles on graphene, which will not damage the original graphene lattice, enhancing the photocurrent [26,27,28]. Moreover, 1D/2D high-speed transistors are obtained through vdW integration of 1D core-shell nanowires and 2D graphene, which has a high cut-off frequency [29,30,31,32]. Based on this progress, in this paper, we propose combining 1D CNTs and 2D GeSe with vdW interaction and explore their electronic properties through first-principles calculations. We aim to provide a theoretical proposal for 1D/2D integration through CNTs and a GeSe monolayer, which has potential applications in the field of optoelectronic devices.

## 2. Computational Method and Model

Our first-principles calculations used the Vienna ab initio simulation package (VASP) [33]. Based on density functional theory (DFT), a plane wave basis expanded the CNT/GeSe hybrid wave function. To represent the interaction of exchange and correlation between the electrons, the Perdew, Burke, and Ernzerhof (PBE) function in the framework of a generalized gradient approximation was used [34,35,36]. To ensure sufficient accuracy, we found that a cut-off energy of 450 eV was satisfactory for the convergence standards. The energy convergence was 10^−6^ eV, while a force of 0.01 eV/Å on each atom was sufficient for the calculations. In the structural relaxation and self-consistent calculation, we set a Monkhorst-Pack grid of *k* points of 8 × 5 × 1 [37] for sampling. When calculating the density of states (DOS) and optical properties, we used a denser Monkhorst-Pack grid of 16 × 10 × 1 *k*-point sampling. For the simulation of a heterostructure between 1D and 2D materials, vdW interaction is especially important. Our simulations adopted semi-empirical dispersion-corrected D3 (DFT-D3) [38] to represent the weak interaction between CNTs and the GeSe monolayer.

**Figure 1:**
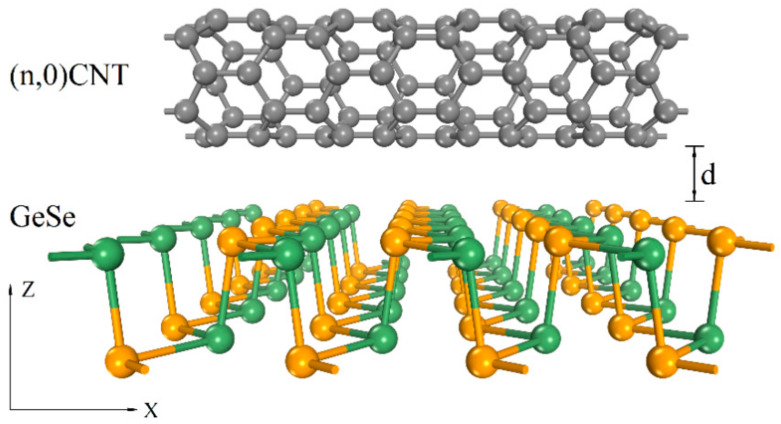
Schematic diagram of the side view of different zigzag CNTs (*n*,0) (*n* = 5–11) on monolayer GeSe. C, Ge, and Se atoms are represented by gray, green, and orange spheres, respectively. *d* is the interfacial spacing between the CNT and monolayer GeSe.

We selected a series of zigzag (*n*,0)CNTs (*n* = 5–11) with an axial length of 4.26 Å to form a composite structure with monolayer GeSe. To build a reasonable 1D/2D model, we first enlarged the unit cell of monolayer GeSe to a 1 × 5 supercell (4.25 Å × 19.95 Å). Based on this supercell, we placed the CNT above the GeSe monolayer along the *x*-axis. In our model, there was only a 0.2% lattice mismatch. We set 28 Å along the *z*-axis as the vacuum layer, which avoids interaction between the adjacent supercells. Figure 1 shows the schematic structural model. To better present the schematic model, we enlarged the indicated lattice constant four times along the *x*-axis.

In our optical calculations using the VASP code, the frequency-dependent dielectric matrix after the electronic ground state was determined [39]. As suggested in [39], the imaginary part can be determined by a summation of the empty states using the following formula:

$$\varepsilon_{2}\left(\omega \right)=\frac{4\pi^{2}e^{2}}{\Omega }\underset{q\to 0}{\lim}\frac{1}{q^{2}}\underset{c,v,k}{\sum }2w_{k}\delta \left(\varepsilon_{ck}-\varepsilon_{vk}-\omega \right)\times (u_{ck}+e_{\alpha q}|u_{vk})(u_{ck}+e_{\beta q}|u_{vk})$$

where the indices *c* and *v* refer to the conduction and valence band states, respectively. In [39] it is stated that $u_{ck}$ is the periodic cell part of the orbitals at the *k*-point *k*, while the *k*-point weights, *w_k_*, are defined such that their sum is one. In addition, in [39], the real part of the dielectric tensor, $\varepsilon_{1}\left(\omega \right)$, is obtained using the usual Kramers–Kronig transformation:

$$\varepsilon_{1}\left(\omega \right)=1+\frac{2}{\pi }P\int_{0}^{\infty }\frac{\varepsilon_{2}\left(\omega^{\prime }\right)\omega^{\prime }}{\omega^{\prime 2}-\omega^{2}+i\eta }d\omega^{\prime }$$

where *P* denotes the principal value while η is an infinitesimal number. In addition, the number of empty bands in the above calculations is twice that of the self-consistent calculations for total energies.

## 3. Results and Discussion

### 3.1. Configurations and Stability of 1D/2D Heterostructures

**Figure 2:**
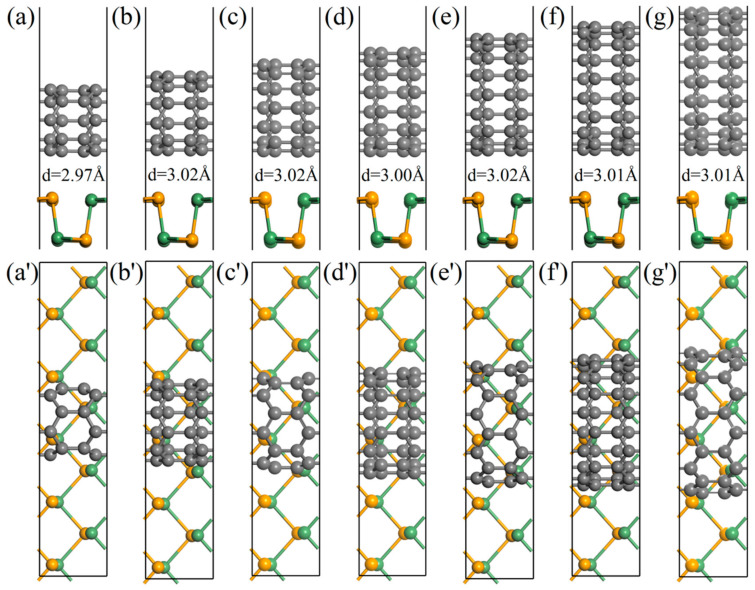
Optimized structures of different CNTs on monolayer GeSe: (**a**–**g**) and (**a′**–**g′**) are side and top view of (*n*,0) CNTs/GeSe (*n* = 5–11), respectively. *d* is the equilibrium spacing between the top Se atomic layer and the annotated wall. Gray, orange, and green spheres represent C, Se, and Ge atoms, respectively.

In this work, six types of zigzags (*n*,0)CNTs (*n* = 5–11) with diameters ranging from 3.92 to 7.83 Å were simulated. It is critical that the 1D CNT and 2D GeSe form a stable composite structure. Figure 2 indicates the optimized configurations of (*n*,0)CNT (*n* = 5–11) on 2D GeSe. From the perspective of the geometrical structure, CNT/GeSe hybrids maintain their original structure. In (*n*,0)CNT/GeSe (*n* = 5–11) with optimized structures, the average C–C bond length of the CNT changes little compared to their components, varying between 0.001 to 0.003 Å. Due to compatibility, the average GeSe bond length in GeSe has only minor variations, ranging from 0.006 to 0.009 Å. The interfacial spacing between the top Se atom of monolayer GeSe and the C atom in a CNT ranges from 2.97 to 3.02 Å (see Table 1). According to previous studies [40,41,42], large interlayer spacing implies weak interaction between GeSe and the CNTs. The calculated formation energy is estimated using the following formula:

(1) $$E_{f}=E_{CNT/GeSe}-E_{CNT}-E_{GeSe}$$

where $E_{CNT/GeSe}$, $E_{CNT}$, and $E_{GeSe}$ are the total energies of the CNT/GeSe heterostructure, CNT, and the monolayer GeSe, respectively.

According to the above definition of formation energy, when *E_f_* is negative, the system tends to be stable. As indicated in Table 1, the negative formation energy of all calculated 1D/2D CNT/GeSe combinations implies the stability of our proposed heterostructures. Our results indicate that the formation energies decrease with increasing CNT diameter. The decreasing formation energy is related to the increased contact area between CNTs and 2D GeSe. In addition, the interlayer interaction of the CNT/GeSe composite structure is reflected by the charge transfer between the CNT and GeSe. We conducted a Bader charge analysis to explore the charge transfer between CNTs and GeSe (see Table 1). A positive Bader charge value indicates that electrons are gained, while a negative value indicates that electrons are lost. Table 1 shows a certain number of electrons are transferred from the 2D GeSe to the CNTs. There are small fluctuations in the amount of charge transfer in (*n*,0)CNT/GeSe (*n* = 5–11), ranging between 0.0159 e to 0.0490 e. The small amount of charge transfer suggests weak interaction between the CNTs and the GeSe monolayer.

**Figure 3:**
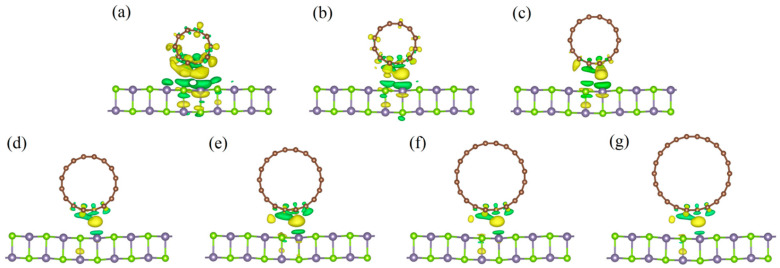
Charge density differences for CNT(*n*,0)/GeSe with *n* = (**a**) 5, (**b**) 6, (**c**) 7, (**d**) 8, (**e**) 9, (**f**) 10, and (**g**) 11. The isovalue is set to 0.0015 *e*/Å^3^. The yellow and green regions represent charge loss and gain of electrons, respectively.

Based on the analysis of the charge density difference, the charge transfer and redistribution at the interface in these hybrids can be evaluated (as shown in Figure 3) by the following relationship:

(2) $$\Delta \rho =\rho_{CNT/GeSe}-\rho_{CNT}-\rho_{GeSe}$$

where $\rho_{CNT/GeSe}$, $\rho_{GeSe},$ and $\rho_{CNT}$ are the charge densities of CNT/GeSe, CNT, and monolayer GeSe, respectively. As Figure 3 indicates, the redistributed charge is visible due to the interaction between the 1D CNTs and 2D GeSe.

As shown in Figure 3, at the interface between GeSe and the CNTs, the charge is transferred from the GeSe to the CNTs. Though the amount of charge transfer is very small, the interaction between CNTs and 2D GeSe can be validated. Moreover, the amount of charge transfer in the (5,0)CNT/GeSe is larger than that in other composites. This is because the interlayer distance between the GeSe and (5,0)CNT is 2.97 Å, which is smaller (as shown in Table 1) than in other hybrid structures.

### 3.2. Band Structure and Density of States

**Figure 4:**
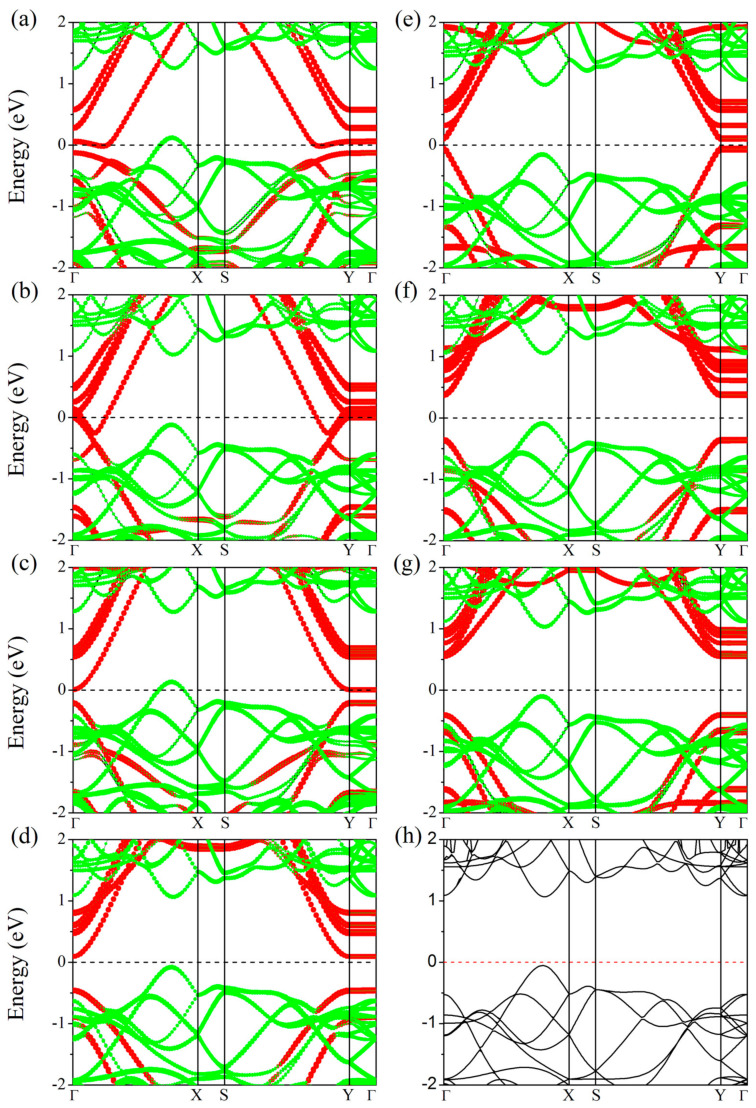
Projected band structures of (*n*,0)CNT/GeSe hybrids with *n* = (**a**) 5, (**b**) 6, (**c**) 7, (**d**) 8, (**e**) 9, (**f**) 10, and (**g**) 11. The red and green circle size denotes the weight of the CNT and monolayer GeSe in the CNT/GeSe configuration band structure. (**h**) is the band structure of pristine 2D GeSe calculated for comparison. A horizontal dashed line represents the Fermi level.

To explore the electronic properties of CNT/GeSe hybrids, we calculated their band structures and DOS. As shown in Table 1, CNT(5,0) and CNT(6,0) are metals while CNT(*n*,0) (*n* = 7–11) are semiconductors. Our results using first-principles calculations are consistent with previous reports in the literature [5]. As shown in Figure 4h, the obtained bandgap of monolayer GeSe is 1.14 eV with our PBE calculation, which is the same as a previous report [43]. As shown in Figure 4a–c and Table 1, (*n*,0)CNT/GeSe (*n* = 5–7) all have bandgaps of zero, while the bandgaps of (*n*,0)CNT/GeSe (*n* = 8–11) are 0.21 eV, 0.16 eV, 0.47 eV, and 0.59 eV. This indicates that CNT/GeSe heterojunctions with small diameter CNTs are metallic. As the diameter of the CNTs increases, the bandgaps of our proposed (*n*,0)CNT/GeSe (*n* = 8–11) heterojunctions gradually increase. However, the bandgap of (9,0)CNT/GeSe is smaller than those of (8,0)CNT/GeSe and (10,0)CNT/GeSe. This is because the bandgap of the (9,0)CNTs/GeSe heterojunction is mainly determined by the energy level of the bands in the (9,0)CNT. In contrast, in (8,0)CNT/GeSe and (10,0)CNT/GeSe heterojunctions, the bandgap is a subtraction between the energy level of the conduction band minimum (CBM) in the corresponding CNTs and the energy level of the valence band maximum (VBM) in the GeSe monolayer. As a result, the bandgap of (9,0)CNT/GeSe is smaller than those of (8,0)CNT/GeSe and (10,0)CNT/GeSe. As the diameter of CNTs increases, the bandgaps of our proposed (*n*,0)CNTs/GeSe (*n* = 8–11) heterojunctions gradually increase. The projected energy band shown in Figure 4 confirms that the CNTs mainly provide the bands near the Fermi surface in the band structure of a CNT/GeSe heterojunction. In other words, whether (*n*,0)CNT/GeSe (*n* = 5–11) heterojunctions composed of (*n*,0)CNTs (*n* = 5–11) and 2D GeSe are metals or semiconductors is mainly determined by the conduction bands near the Fermi level. Due to the weak van der Waals interaction between CNTs and 2D GeSe, we can use CNTs with different diameters to obtain suitable bandgaps in CNT/GeSe heterojunctions with the variation of the band structure in CNTs.

**Figure 5:**
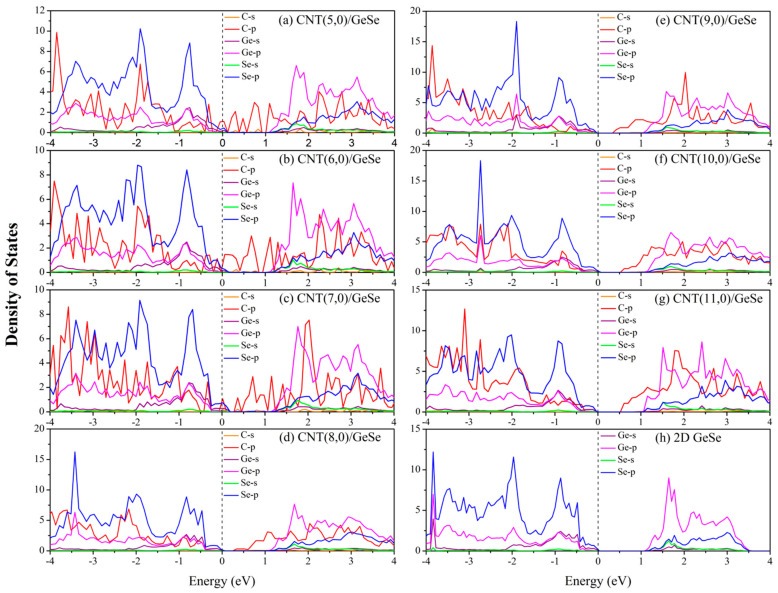
PDOS of CNT(*n*,0)/GeSe hybrids with *n* = (**a**) 5, (**b**) 6, (**c**) 7, (**d**) 8, (**e**) 9, (**f**) 10, (**g**) 11, and a (**h**) monolayer GeSe. The Fermi level is set to zero.

In Figure 5, we show the partial DOS (PDOS) of (*n*,0)CNT/GeSe (*n* = 5–11) and monolayer GeSe. Compared with the PDOS of monolayer GeSe, the PDOS of GeSe in the heterojunction is the same as that of the monolayer GeSe. The total DOS of the CNT/GeSe heterojunction can be viewed as a superposition of the DOS in the CNT and in the 2D GeSe. This further proves that there is a weak vdW interaction between the CNTs and 2D GeSe. From the perspective of the PDOS, the conduction band near the Fermi surface of the CNT/GeSe heterojunction is provided by the CNT and GeSe together. In contrast, the valence band is mainly provided by the 2*p* orbital of carbon atoms. Aside from the 2*p* orbital of carbon atoms, the orbital DOS of the other elements is unchanged. It is the 2*p* orbital of carbon that determines the top position of the valence bands, thus, affecting the band structure of the heterojunction. As shown in Figure 5a–c, the 2*p* orbital of carbon in (*n*,0)CNT/GeSe (*n* = 5–7) passes through the Fermi level, so those heterojunctions are metallic. The 2*p* orbital of carbon in (8,0)CNT/GeSe and (9,0)CNT/GeSe is closer to the Fermi surface than that in the (10,0)CNT/GeSe and (11,0)CNT/GeSe heterojunctions, which leads to smaller bandgaps in (8,0)CNT/GeSe and (9,0)CNT/GeSe heterojunctions than those in (10,0)CNT/GeSe and (11,0)CNT/GeSe heterojunctions.

**Figure 6:**
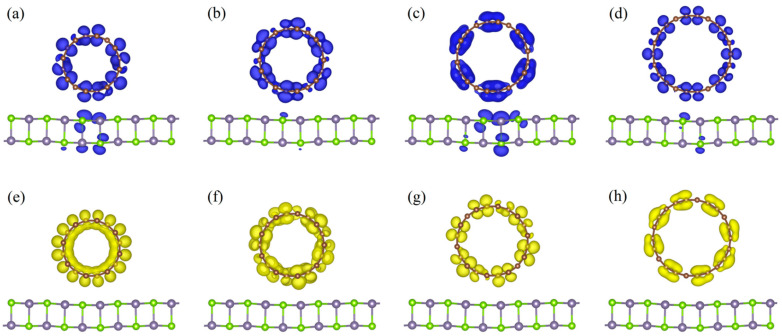
Maps of the hole and electron density distributions for (**a**–**d**) VBM and (**e**–**h**) CBM of CNT(8,0)/GeSe, CNT(9,0)/GeSe, CNT(10,0)/GeSe, and CNT(11,0)/GeSe with an isovalue of 0.007 e/Å^3^, respectively. Blue and yellow regions denote the hole and electron density distributions of the VBM and CBM, respectively. Brown, cyan, and purple spheres represent C, Se, and Ge atoms, respectively.

To further confirm the distribution of electronic states near the Fermi surface of the CNT/GeSe, in Figure 6 we show the electronic state distribution of (*n*,0)CNT/GeSe (*n* = 8–11). The CNT and GeSe provide the holes at the top of the valence band, with most holes provided by the CNT. In comparison, the electrons at the bottom of the conduction band are provided by the CNT alone. From the previous discussion of the PDOS, the 2*p* orbital of carbon contributes electrons at the bottom of the conduction band. Therefore, the electronic properties in CNT/GeSe heterojunctions are mainly influenced by CNTs with varying tube diameters. In other words, we found that the bandgap of our studied heterojunctions can be tuned by varying the tube diameter of the CNTs. Our calculated results predict that the bandgap of the heterojunction is smaller than that of monolayer GeSe, which is beneficial for optical absorption.

### 3.3. Optical Absorption Properties

**Figure 7:**
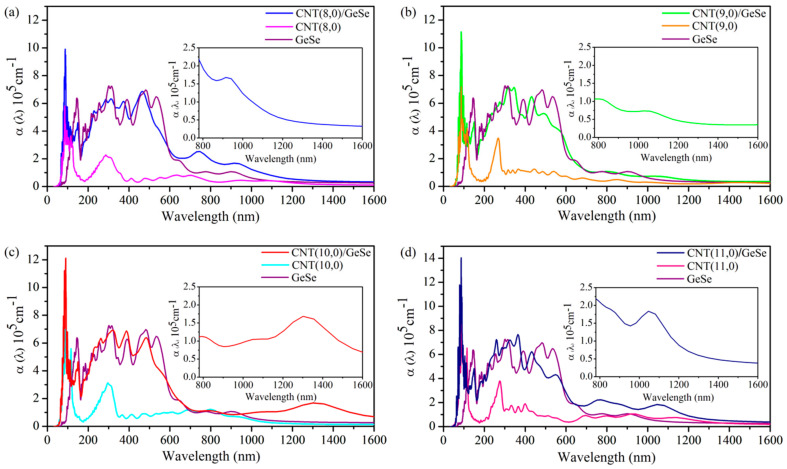
Optical absorption coefficient, α, of (**a**) (8,0)CNT/GeSe, monolayer GeSe, and (8,0)CNT; (**b**) (9,0)CNT/GeSe, monolayer GeSe, and (9,0)CNT; (**c**) (10,0)CNT/GeSe, monolayer GeSe, and (10,0) CNT; and (**d**) (11,0)CNT/GeSe, monolayer GeSe, and (11,0)CNT at the zigzag direction.

To evaluate the optical absorption properties of CNT/GeSe heterojunctions, we utilized the following formula to assess the optical coefficient:

(3) αω=2ωε12ω+ε22ω−ε1ω12

where $\varepsilon_{1}\left(\omega \right)$ and $\varepsilon_{2}\left(\omega \right)$ are the real and imaginary parts of the complex dielectric function, respectively. In Figure 7, we present the calculated optical absorption coefficient, α(ω) of a monolayer GeSe, pure CNT, and CNT/GeSe hybrids. According to previous studies [16], GeSe reportedly had good optical absorption properties. By combining 2D GeSe with CNTs, the bandgap of the hybrid system is smaller than that of 2D GeSe, which is helpful for the separation of photogenerated electrons and holes. Figure 7 shows the optical absorption of our studied CNT/GeSe hybrids. For comparison, the calculated optical absorption of a GeSe monolayer and CNTs are plotted together. The results indicate that (*n*,0)CNT/GeSe (*n* = 8–11) all have good optical absorption in the visible light region, which has a high optical absorption peak of about 6 × 10^5^ cm^−1^. In the infrared region, the optical absorption coefficients of (*n*,0)CNT/GeSe (*n* = 8–11) are significantly enhanced compared to those of GeSe. The optical absorption peaks of (8,0)CNT/GeSe and (11,0)CNT/GeSe in the infrared region reach 2 × 10^5^ cm^−1^. The optical absorption peak of (10,0)CNT/GeSe is close to 2 × 10^5^ cm^−1^. In the ultraviolet region, the light absorption of the CNT(*n*,11)/GeSe (*n* = 8–11) composite is greatly enhanced compared to the corresponding components of GeSe and CNT. Our results prove that the optical absorption of the combined structure of (*n*,0) CNTs (*n* = 8–11) and 2D GeSe is substantially enhanced compared with that of 2D GeSe and (*n*,0) CNTs (*n* = 8–11).

**Figure 8:**
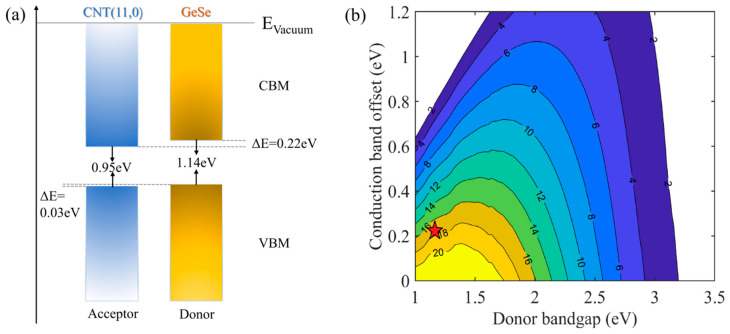
(**a**) Schematic representation of the band alignment of the (11,0)CNT/GeSe heterostructure. (**b**) Contour plot of power conversion efficiency (%) for the (11,0)CNT/GeSe heterostructure.

According to the theory suggested by Scharber et al., [44], the PCE *η* of CNT/GeSe can be described as follows:

(4) $$\eta =\frac{J_{SC}V_{OC}\beta_{FF}}{P_{SOLAR}}=\frac{0.65\left(E_{g}^{d}-\Delta E_{c}-0.3\right)\int_{E_{g}^{d}}^{\infty }\frac{P\left(\hbar \omega \right)}{\hbar \omega }d\left(\hbar \omega \right)}{\int_{0}^{\infty }P\left(\hbar \omega \right)d\left(\hbar \omega \right)}$$

where the fill factor ($\beta_{FF}$) is 0.65. The maximum open-circuit voltage ($V_{OC})$ is estimated by $\left(E_{g}^{d}-\Delta E_{c}-0.3\right)$, where $E_{g}^{d}$ is the donor bandgap. $\Delta E_{c}$ is the conduction band offset (CBO) between the donor (GeSe) and acceptor (CNT). $P\left(\hbar \omega \right)$ is the AM1.5 solar energy flux at the photon energy (ħω). The integral in the numerator is the short circuit current ($J_{SC}$) performed by applying an external quantum efficiency limit of 100%, and the integral in the denominator in Equation (4) is the incident solar radiation ($P_{SOLAR}\;$= 1000 Wm^−2^). As mentioned in our analysis of the band structure, the donor layer is the GeSe monolayer with a bandgap of 1.14 eV, while the value of the CBO in the (10,0)CNT/GeSe heterostructure is 0.48 eV. We found that the PCE of the (10,0)CNT/GeSe heterostructure reaches 11.04% following the calculated definition. To achieve a higher PCE by combining CNTs and GeSe, we further obtain the optimized structure of (11,0)/GeSe and the corresponding band structure using the same simulation method. As shown in Figure 8, the (11,0)CNT/GeSe heterostructure has type-II band alignment. The type-II heterostructure facilitates the separation of photogenerated carriers and holes. The value of the CBO in the (11,0)CNT/GeSe heterostructure is 0.22 eV. The PCE of the (11,0)CNT/GeSe heterostructure reaches 17.29%. The obtained high PCE in the (11,0)CNT/GeSe heterostructure is comparable to that in bilayer phosphorene/MoS_2_ (16%–18%) [45] and GeSe/SnS (18%) [46] heterostructures.

## 4. Conclusions

We proposed new types of CNT/GeSe heterojunctions by combining a 1D CNT (*n* = 5–11) and 2D GeSe, and calculated their electronic and optical properties based on DFT. Our calculations show that CNT/GeSe (*n* = 5–11) are stable through weak vdW interactions. Among the structures, (*n*,0)CNT/GeSe (*n* = 5–7) exhibit metallic properties, while (*n*,0)CNT/GeSe (*n* = 8–11) have small bandgaps, lower than 0.8 eV. Due to their small bandgaps, (*n*,0)CNT/GeSe (*n* = 8–11) have excellent optical absorption properties, especially in ultraviolet and infrared absorption. The absorption coefficient of (*n*,0)CNT/GeSe (*n* = 8–11) in the ultraviolet region can reach the order of 10^5^ cm^−1^. In particular, we found that the (11,0)CNT/GeSe heterostructure exhibits type-II band alignment and a high PCE of 17.29%. Our study implies that 1D/2D GeSe/CNT heterostructures have potential applications in photoelectronics and photodetection.

## Figures and Tables

**Table 1:** Diameter and bandgaps of (*n*,0) CNTs (*E_g_*) (*n* = 5–11) and formation energy, *E_f_*, bandgap, *E_g_*^a^, and interfacial spacing, *d*, of optimized CNT/GeSe hybrids. Bader charge: the positive value indicates gained electrons, while a negative value reveals lost electrons.

Hybrid	Diameter (Å)	*E_g_* (eV)	*E_g_*^a^ (eV)	*E_f_* (eV)	*d* (Å)	Bader Charge (e)	
						GeSe	CNT
CNT(5,0)/GeSe	3.92	0	0	−3.517	2.97	−0.1098	0.1098
CNT(6,0)/GeSe	4.70	0	0	−3.959	3.02	−0.0490	0.0490
CNT(7,0)/GeSe	5.48	0.1746	0	−4.078	3.02	−0.0280	0.0280
CNT(8,0)/GeSe	6.27	0.5908	0.2112	−4.273	3.00	−0.0204	0.0204
CNT(9,0)/GeSe	7.05	0.1568	0.1643	−4.283	3.02	−0.0159	0.0159
CNT(10,0)/GeSe	7.83	0.7222	0.4669	−4.513	3.01	−0.0280	0.0280
CNT(11,0)/GeSe	8.59	0.9519	0.5924	−4.709	3.01	−0.0174	0.0174

## Data Availability

The data presented in this study are available on request from the corresponding author.
